# Prevalence of cardiovascular abnormalities in obese adults referred for bariatric weight loss surgery

**DOI:** 10.1186/1532-429X-17-S1-P188

**Published:** 2015-02-03

**Authors:** Tendoh Timoh, Rishikesh Dalal, Saleem Ali, Manish Parikh, Monvadi B Srichai

**Affiliations:** Cardiology, Medstar Georgetown University Hospital, Washington, DC USA; Cardiology, Medstar Washington Hospital Center, Washington, DC USA; NYU Langone Medical Center, New York, NY USA

## Background

Obesity is associated with increased risk of death that may be related to underlying structural and functional changes in the heart. Cardiac MR (CMR) can provide detailed structural and functional information about the heart and vasculature that may improve our understanding of obesity related cardiac morbidity and mortality.

## Methods

A cohort of 77 obese patients referred for stress CMR for evaluation of dyspnea prior to weight-loss surgery (WLS) were identified from our research registry. All patients underwent standard vasodilator stress perfusion protocol with late gadolinium enhancement (LGE) imaging on a 1.5T MR system. We collected demographic and CMR data including ventricular volumes, global and regional functional indices, and results of stress and LGE imaging. Comparison of CMR was made to previously derived and universally accepted normative data. The goal will be to assess these parameters 1 year post WLS and analyze the structural, functional and microvascular changes.

## Results

Please see table 1.

**Table 1:** Summary data of LV and RV indices compared to normative derived data* (mean ± SD, 95% confidence interval)

Categories	Obese Cohort (n=77)	Normal* (n=120)
Age (years)	48.7 ± 2.5 (22, 72)	20 - 79 years (equally split in each decile)
Gender	51 female (66%)	60 female (50%)
Body Mass Index (kg/m2)	48.1 ± 1.8 (35.5, 72)	
Body Surface Area (Dubois, m2)	2.3 ± 0.1 (1.8, 3)	
LVEDV (mL)	165 ± 9.1 (95, 344)	142 ± 21 (102, 183)
LVEDV/BSA (mL/m2)	71.3 ± 2.9 (45, 127)	78 ± 8.8 (60, 95)
LVESV (mL)	64.6 ± 7 (23, 254)	47 ± 10 (27, 68)
LVESV/BSA (mL/m2)	27.7 ± 2.5 (12, 94)	26 ± 5.1 (16, 36)
LVEF (%)	62.4 ± 1.9 (26, 83)	67 ± 4.6 (58, 76)
RVEDV (mL)	174 ± 10.4 (76, 295)	144 ± 23 (98, 190)
RVEDV/BSA (mL/m2)	75 ± 3.4 (42, 123)	78 ± 11 (57, 99)
RVESV (mL)	72.2 ± 5.9 (23, 168)	50 ± 14 (22, 78)
RVESV/BSA (mL/m2)	31.1 ± 2.1 (12, 62)	27 ± 7 (13, 41)
RVEF (%)	59.1 ± 1.6 (40, 75)	66 ± 6 (54, 78)
Abnormal LV diastolic function	> 21 %	
Ischemia on stress perfusion	38/77 (49%)	
• Subendocardial ischemia	34/77 (44%)	
LV mass (grams)	145 ± 10.6 (70, 276)	127 ± 19 (90, 164)

*Comparison made to published age, gender, and height matched normal weight controls.

LV, left ventricle; RV, right ventricle; EDV, end-diastolic volume; ESV, end-systolic volume; EF, ejection fraction.

## Conclusions

Multiple cardiovascular abnormalities are noted in severely obese patients including LV and RV dilation and microvascular ischemia that may contribute to the increased cardiovascular morbidity and mortality observed in this population.

## Funding

Empire Clinical Research Investigator Program (ECRIP).

